# Caprine demineralized bone matrix (DBMc) in the repair of non-critical bone defects in rabbit tibias. A new bone xenograft^1^

**DOI:** 10.1590/s0102-865020200080000001

**Published:** 2020-09-04

**Authors:** Felipe Rocha dos Santos, Bruno Watanabe Minto, Sidney Wendell Goiana da Silva, Livia de Paula Coelho, Pedro Paulo Rossignoli, Jose Sergio Costa, Mario Taba, Luis Gustavo Gosuen Gonçalves Dias

**Affiliations:** I Fellow Master degree, Postgraduate Program in Veterinay Surgery, Universidade Estadual Paulista (UNESP), Jaboticabal-SP, Brazil. Conception and design of the study; acquisition, analysis and interpretation of data, manuscript preparation and writing, final approval.; II Full Professor, Department of Veterinary Clinics and Surgery, School of Agricutural Sciences and Veterinary Medicine, UNESP, Jaboticabal-SP, Brazil. Conception and design of the study, analysis and interpretation of data, manuscript preparation and writing, final approval.; III Full Professor, School of Medicine, Universidade Federal do Ceará (UFCE), Sobral-CE, Brazil. Prepared and supplied the tested biomaterial.; IV Fellow PhD degree, Postgraduate Program in Veterinay Surgery, UNESP, Jaboticabal-SP, Brazil. Conception and design of the study; acquisition, analysis and interpretation of data.; V Associate Professor, School of Dentistry of Ribeirao Preto (FORP), Universidade de São Paulo (USP), Ribeirao Preto-SP, Brazil. Performed the microtomographic analyzes of the samples.

**Keywords:** Bone Transplantation, X-Ray Microtomography, Bone Substitutes, Goats, Tibia, Rabbits

## Abstract

**Purpose:**

To evaluate the use of demineralized bone matrix of caprine origin in experimental bone defects of the tibia in New Zealand rabbits.

**Methods:**

Fragments of the tibia diaphysis were collected aseptically from clinically healthy goats. The bones were sectioned into 1 cm fragments and stored at -20°C for subsequent hydrochloric acid (HCL) demineralization. A 70 mg portion of DBMc was used to fill the experimental bone defects. Twenty-four female adult New Zealand rabbits were divided into 2 groups: the MG (matrix group, left tibia) and CG (control group, right tibia). Additionally, they were separated into 4 groups with 6 animals, according to the period of analysis (15, 30, 60 and 90 days postoperatively). Using microCT, volumetric parameters were evaluated: bone volume, relationship between bone volume and total volume, bone surface area, relationship between bone surface area and total volume, number of trabeculae, trabecular thickness and trabecular separation.

**Results:**

There was a statistically significant difference (P<0.05) between groups considering bone volume (BV) and bone:total volume (BV/TV), on 15, 30 and 90 days postoperatively. Control group showed a statistically significant superiority (P < 0.05) considering the mean of the variables bone surface (BS), number of trabeculae (Tb.N) and between bone surface and total volume (BS/TV) at 15 and 90 days.

**Conclusions:**

Caprine demineralized bone matrix was safe and tolerable. No signs of material rejection were seen macroscopically. It is an alternative for the treatment of bone defects when autologous graft is not available or in insufficient quantities.

## Introduction

Bone grafting is a fundamental part of reparative surgery^1^. Despite the development of alternatives, autologous cancellous bone graft is still considered the gold standard to assist the treatment of bone fractures and nonunions^2^. However, the use of bone graft has some limitations such as donor site morbidity and grafts quantity^3-5^, which stimulate a growing demand for ideal bone substitutes^1^.

Demineralized bone matrix (DBM) is one of the best known clinically established bone grafts, and several studies have demonstrated its potential^5^. DBM is produced by mineral acid extraction of cortical bones, and consists mainly of collagenous matrix, and to a lesser extent growth factors and bone morphogenetic proteins^6-7^. Amongst its advantages are osteoinduction capacity, osteoconduction and the ease with which large amounts of matrix can be obtained^1,8^.

Demineralized bone matrix xenograft is most commonly derived from cortical and cancellous bone of bovine origin^5,8,9^, from pigs, and has low inflammatory tissue reaction and trabecular bone formation^10^.

Caprine demineralized bone matrix (DBMc) is a potential bone substitute, which despite its xenogenic origin, has the advantage of providing a significant amount of bone tissue from a single donor, enabling the creation of bone banks^11,12^. Studies involving goat bone graft is scarce, thus the objective of this study was to evaluate macroscopic inflammatory and bone healing after implantation of DBMc in surgically induced tibial defects in rabbits. Our hypothesis was that the DBMc-treated group had no macroscopic signs of implant rejection, greater bone volume, bone surface, and number of trabeculae compared to the untreated group.

## Methods

### *Animals and experimental design*

Twenty-four female adult New Zealand rabbits were used following a protocol approved by the Animal Use Ethics Commission (CEUA) of Universidade Estadual Paulista (UNESP), Campus Jaboticabal (Protocol number 007310/17).

The MG (matrix group) was represented by the left tibia of each rabbit (n=24) and received the implant of DBMc, while the CG (control group) was compounded by the right tibia (n=24), in which no biomaterial was used. Later, they were separated into 4 groups with 6 animals (n=12), according to the period of analysis (15, 30, 60 and 90 days postoperatively).

### *Demineralized bone matrix*

Caprine Demineralized Bone Matrix (DBMc) was collected and processed following the protocol previously described^8^. Seventy (70) mg of DBMc was required to fill the experimental bone defects.

### *Sedation and surgical procedure*

**Figure 1:**
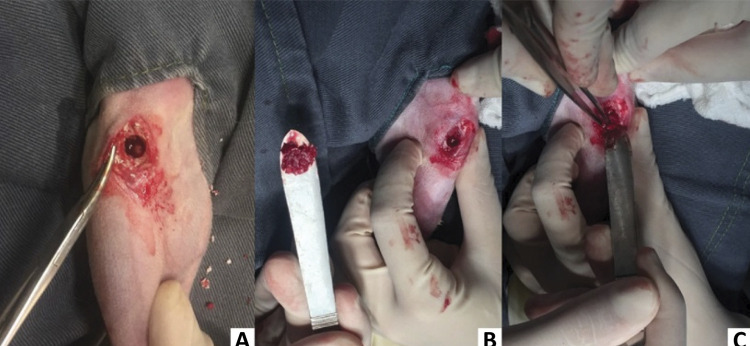
Intraoperative images demonstrating the application of caprine Demineralized Bone Matrix (DBMc) in the proximal tibial region. (A) Non-critical bone defect has been created; (B) DBMc was moistened with bone intramedullary blood; and (C) implantation of DBMc.

All rabbits were anesthetized with 0.1 mg kg of acepromazine (IM), 5 mg/kg of tramadol (IM) and a combination of tiletamine/zolazepam (20 mg/kg [IM]). Both pelvic limbs were aseptically prepared. A skin incision was made in the medial face of the proximal right and left tibia of each animal. The medial cortex of the proximal tibia was exposed, and a 6 mm-diameter circular bone defect was created in both tibias (Fig. 1A). For the left tibia (MG), the particulate DMBc was moistened with bone intramedullary blood (Fig. 1B) before implantation (Fig. 1C). The surgical wound was closed routinely.

All animals received 10 mg/kg of enrofloxacin (SC/SID/5 days), 4 mg/kg of tramadol hydrochloride (IM/BID/5 days) and 1.1 mg/kg of flunixin meglumine (IM/SID/3 days). The surgical wounds were observed for healing and possible complications.

### *Sample collection and microtomographic parameters analysis*

Six animals from each group were properly euthanized at pre-established periods. Two cm bone fragment was sectioned, including the area of interest of the study. The samples were placed in collection flasks containing 10% buffered formaldehyde for 48 hours for fixation, transferred to 70-alcohol containers and individually identified, according to time of collection and whether or not DBMc had been used.

The microtomography scan (SkyScan® 1172 microtomograph, Bruker, Belgium) was performed and microtomographic reconstruction of bone samples was made by Nrecon® software (NRecon® Software, Bruker, Belgium).

Volumetric parameters were evaluated: Bone volume, relationship between bone volume and total volume, bone surface, relationship between bone surface and total volume, number of trabeculae, trabecular thickness and trabecular separation.

For the volumetric analysis, an axis of interest (sagittal) was chosen and the CT-Analyzer® software (CT-Analyzer® Software, Bruker, Belgium) specific for three-dimensional analyses was used. Data Viewer® software (Data Viewer® software, Bruker, Belgium) was used for two-dimensional (2D) visualization and evaluation of sagittal, coronal and transaxial microtomography images (SkyScan®, Version 1.4.4 64-bit).

From the generated images, the CT-Analyzer® software created a text file of the trabecular bone filling pattern (number, thickness and separation of the trabeculae) in the tibial defects, production of bone tissue (bone volume, bone surface area and percentage of bone volume formed), with and without xenograft application, allowing the relationship between the application of caprine demineralized bone matrix and bone proliferation to be determined.

### *Statistical analysis*

Statistical analysis was performed using the R software (Software R - version 3.5.1, GNU Project). To select the most appropriate statistical test, homoscedasticity (Levene test) and normality (Cramer-von Mises test) were assessed. Statistical analysis was performed by applying parametric tests. To compare the results obtained in the control group (CG), the matrix group (MG) and the different euthanasia times (15, 30, 60 and 90 days), the ANOVA analysis of variance test was used. The means between control group and matrix at each euthanasia time were then compared using the T student test, considering a significance level of 95% (p <0.05).

## Results

All animals were able to ambulate and were with good weight bearing in both operated limbs since immediately after anesthetic recovery. No signs of inflammation, self-mutilation or seroma indicating surgical site infection and / or graft rejection were observed.

### *Microtomographic analysis*

**Figure 2:**
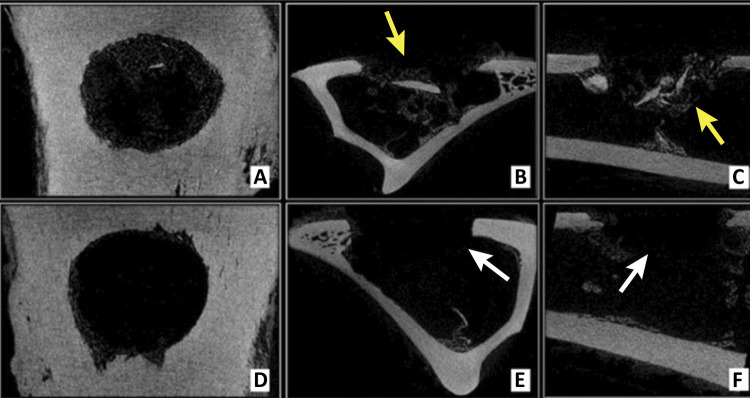
Microtomographic images at 15 days postoperatively, from Data Viewer® software, in three axes (sagittal, coronal and transaxial - respectively). Control group (A, B and C) and Matrix group (D, E and F). Note cortico-cancellous bone fragments on bone defect in control group (*yellow arrows*), and radiolucent area in the center of bone defect in the test group, representing the presence of DBMc (*white arrows*).

Radiopaque bone fragments were visible in the center of the bone defect (these were believed to be left over from the surgical intervention) on microCT images of the control group at 15days postoperatively (Fig. 2). In the images from matrix group, the center of the bone defect was radiolucent at 15 days due to the presence of the newly implanted demineralized matrix. No significant bone proliferation was seen in any of the groups at this time.

**Figure 3:**
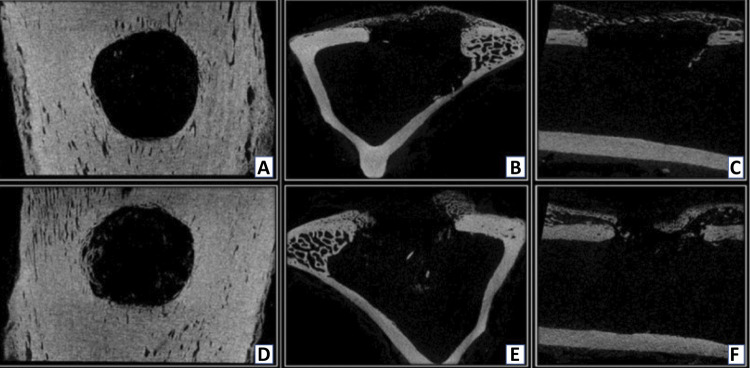
Microtomographic images obtained at 30 postoperative days, using Data Viewer® software, in three axes (sagittal, coronal and transaxial - respectively). Control group (A, B and C) and Matrix group (D, E and F). Note initial bone repair, oriented from the margins to the center of bone defect in both groups. Greater distance between the edges of the newly formed repair tissue can be seen in the MG images (A, B and C) compared to CG (D, E and F).

The microCT images, in the sagital axis, showed radiolucency in the center of the defect of both groups at 30 days (Fig. 3). In the coronal and transaxial axes, early bone repair was visible, from the margins to the center of bone defects. Bone proliferation was good in both groups, but with higher proliferative intensity in the control group samples. The radiopacity of the newly formed bone tissue was less intense when compared to the adjacent original cortical bone at the edges of the analyzed defect.

**Figure 4:**
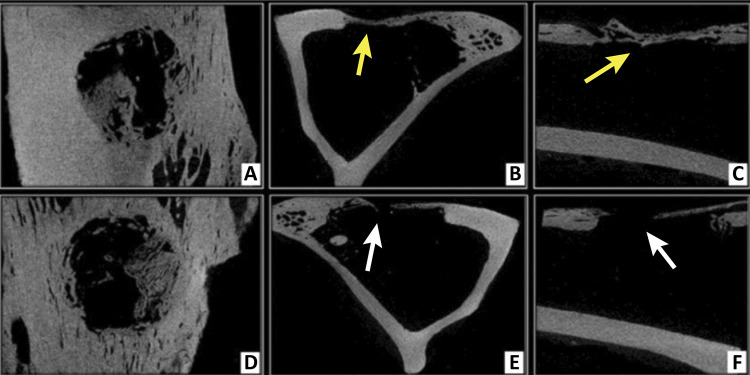
Microtomographic images obtained at 60 days postoperatively, using Data Viewer® software, in three axes (sagittal, coronal and transaxial - respectively). Control group (A, B and C) and Matrix group (D, E and F). Partial filling of the bone defect is seen in both groups, with greater bone volume newly formed in the CG samples (*yellow arrows*) compared to MG (*white arrows*).

On the 60th postoperative day, partial bone filling was observed in the three projections, with greater bone proliferation in the control group samples when compared to the group receiving the demineralized bone matrix (Fig. 4). The bridging tissue had a more radiopaque appearance compared to that observed at 30 days, approaching the original bone density.

**Figure 5:**
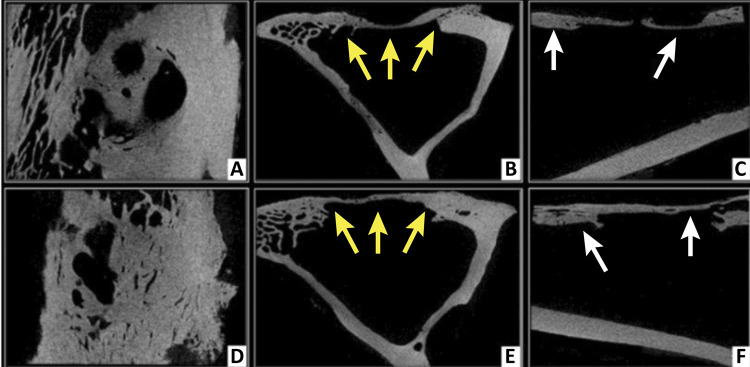
Microtomographic images obtained at 90 postoperative days, using Data Viewer® software, in three axes (sagittal, coronal and transaxial - respectively). Control group (A, B and C) and Matrix group (D, E and F). Subtotal filling of bone defects in both groups, with newly formed bone in a bridging pattern (*yellow arrows*), with similar density to the original cortex of the edges, but less thick (*white arrows*).

At 90 days, the microCT images showed almost complete closure of the bone defects in both groups, in the three axes analyzed (Fig. 5). Bone bridging was thicker and more radiopaque when compared to previous experimental times.

### *Volumetric measurements*

The numerical values of the analyzed variables (Bone Volume, Bone Volume to Total Volume Relationship, Bone Surface area, Bone Surface area to Total Volume Ratio, Number of Trabeculae, Trabecular Thickness and Trabecular Separation) are shown in Table 1.

**Table 1:** Means and standard deviations of volumetric parameters (Bone Volume, Bone Volume to Total Volume Ratio, Bone Surface area, Bone Surface area to Total Volume Ratio, Number of Trabeculae, Trabecular Thickness and Trabecular Separation) of proximal tibial specimens from adult New Zealand rabbits at euthanasia times of 15, 30, 60 and 90 days.

Groups	Bone Volume (BV, mm^3^)	BV/TV (%)	Bone Surface Area (BS, mm^2^)	BS/TV (mm)	Number of Trabeculae (Tb.N, mm)	Trabecular Thickness (Tb.Th, mm)	Trabecular Separation (Tb.Sp, mm)
CG15	2.65 ± 1.56 a	5.39 ± 3.18 a	193.28 ± 55.79 a	3.92 ± 1.14 a	0.73 ± 0.23 a	0.06 ± 0.03a	0.54 ± 0.11a
MG15	0.56 ± 0.46 b	1.14 ± 0.94 b	37.05 ± 18.19 b	0.74 ± 0.37 b	0.11 ± 0.07 b	0.07 ± 0.06a	0.66 ± 0.19a
CG30	5.6 ± 2.54 a	11.39 ± 5.15 a	242.64 ± 92.83 a	5.02 ± 1.88 a	1.39 ± 0.52a	0.07 ± 0.02a	0.6 ± 0.24a
MG30	3.37 ± 2.56 b	6.87 ± 5.21 b	169.28 ± 89.96 a	3.43 ± 1.83 a	0.87 ± 0.57 a	0.06 ± 0.05 a	0.56 ± 0.25 a
CG60	6.27 ± 2.17 a	12.76 ± 4.42 a	196.02 ± 81.05 a	3.97 ± 1.64 a	1.29 ± 0.52 a	0.09 ± 0.03 a	0.95 ± 0.35 a
MG60	5.1 ± 2.01 a	10.38 ± 4.10 a	158.29 ± 63.03 a	3.21 ± 1.28 a	0.99 ± 0.45 a	0.08 ± 0.05 a	0.89 ± 0.15 a
CG90	10.45 ± 2.30 a	21.25 ± 4.67 a	188.82 ± 28.18 a	3.83 ± 0.57 a	1.49 ± 0.32 a	0.13 ± 0.03 a	0.89 ± 0.12 a
MG90	7.08 ± 2.25 b	14.39 ± 4.57 b	130.76 ± 48.06 b	2.65 ± 0.98 b	0.94 ± 0.44 b	0.15 ± 0.03 a	0.97 ± 0.22

*Different letters for animals in the control group and matrix group at the same euthanasia times indicate statistical significance between the means by the Student T test (P <0.05).

BV: Bone Volume

TV: Total Volume

BV / TV: Relationship between bone volume and total volume

BS / TV: Relationship between bone surfasse area and total volume

Tb.N: Number of trabeculae

Tb.Th: Trabecular thickness

Tb.Sp: Trabecular Separation

There was a statistically significant difference (P<0.05) between the mean Bone Volume of the control and matrix groups on days 15, 30 and 90. The relationship between Bone Volume and Total Volume, expressed as a percentage, showed a statistically significant difference (P<0.05) at 15, 30 and 90 days postoperatively. There was a statistical difference between the control and matrix groups, for the variable Bone Surface area (mm^2^), at 15 and 90 days postoperatively (P <0.05).

The relationship between Bone Surface area and Total Volume (mm) showed a statistically significant difference (P <0.05) at euthanasia times of 15 and 90 days.

There was a statistical difference (P<0.05) in parameter Number of Trabeculae (mm) between the control and matrix groups at 15 and 90 days. No statistical difference was found for either the mean three-dimensional variables of Trabecular Thickness (mm) and Trabecular Separation (mm) between the control and matrix groups at euthanasia times (15, 30, 60 and 90 days).

## Discussion

The autologous graft has been the gold standard procedure for bone reconstruction, especially due to its capacity to conduct, induce and generate bone. However, it has been known that autologous grafting has limitation related to donor site morbidity and volume of possible harvesting, leading to an imminent necessity of searching for a suitable and widely available bone graft^13^. Among numerous options of bone substitutes, demineralized bone matrix (DBM) has been considered versatile and extensively used for repairing bone, in humans and veterinary patients^14,15^.

Results of the current study provide the quantification of bone regeneration with or without goat bone xenograft in rabbit tibia by microtomographic measurement. Considering our methodology, the hypothesis that the caprine xenograft is safe when used in bone defects is supported, but the treated group did not present faster bone regeneration than the control group, rejecting the hypothesis that DBMc would end up in an increased bone healing.

Bone xenografts have been studied as a replacement for allograft and autograft by several authors. Most studies used bone of bovine^5,16,17^ and pork origin^18,19^. The present study used caprine demineralized bone matrix in the healing of a non-critical bone defects as an alternative bone substitute.

Studies involving a xenograft from caprine origin are still scarce, and those that have been published so far are conflicting to establish the efficacy of this product. El-Keiey *et al*.^20^ reported a successfully use of autoclaved cortical bones of goats for repairing bone defects induced in the femur of dogs, that showed full recovery of the operated limb after 12 weeks in 80% of them, with no signs of infection or immunological reactions. On the other hand, a xenogenic platelet-rich plasma was tested for healing of bone defects in immunosuppressed rats, and the authors concluded that there was no effect of caprine PRP on bone formation after 1, 2, 6 and 12 weeks^21^. Additionally, other tissues from goats have been studied for application in bone repairing surgeries with apparent good results. Gupta *et al*.^12^ showed a scaffold from decellularized and modified goat lung tissue and demonstrated formation of a biocompatible three-dimensional matrix which potentially improves osteblastic activity, including cell adhesion, growth and proliferation.

It was not possible to establish the age and sex of the donor animals. However, osteoinductive activity of the demineralized bone matrix was observed when implanted at the recipient site, regardless of age and / or gender of the donor animal. These characteristics also showed no influence on the response of the recipient animal^1,22^. Additionally, the size of the particles of the biomaterial has shown to interfere in its ability to improve bone healing^13,20^. According to Dozza *et al*.^13^, the collagen structure present in the demineralized bone matrix can be affected by the size of the particles; fragments of 0.5 and 1 mm seem to provide more efficient and consistent results in relation to cytocompatibility and osteoinduction in vivo, when compared to those <0.5 mm and 1 to 2 mm. Larger particles provide, potentially, less total area for remodeling, taking up space in bone failure and leading to a delay in normal mineralization, similar to that observed in the group that received DBMc, and potential influence on the results obtained^11^. Although particulate biomaterial has been used, no standardized granulometry was established, being considered a limitation of this study.

The lack of a carrier for the demineralized bone matrix implant made manipulation of the biomaterial difficult during surgical implantation and some tissue may have lost into the marrow cavity^5,23^. Pure particulate biomaterial was used to preserve the characteristics of the demineralized bone matrix, since the addition of carrier substances may alter activity^24^. Since there are no reports in the current literature on the implantation of caprine demineralized bone matrix in bone defects, we needed to avoid use of any materials that may increase implant rejection. One of the major complications of xenograft use is tissue incompatibility, resulting in intense and hyperacute inflammatory reaction^4,16^. In this study, no inflammation, self-mutilation or any complications indicating graft rejection or surgical site infection were seen^25^.

Several studies have shown satisfactory results using demineralized bone matrix. In a recent one^9^, it was compared demineralized bone matrix of bovine origin and autogenous graft in a surgically created defects in the radius of rabbits and concluded that the xenogenic graft group had satisfactory bone healing, reducing the morbidity of the donor site when compared to the autograft. Another study^3^, the authors concluded that the use of xenograft alone is not sufficient to accelerate bone healing, as well as it was observed in our study; despite the evident biocompatibility of the xenograft, there was no improvement in bone consolidation when using DBMc alone, without carriers or adjuvant substances.

Computed microtomography allows quantification of the structural properties of hard tissues^26^, and is a noninvasive method of three-dimensional structural analysis^27^. In this study, microtomographic images gave detailed information on the progression of bone repair over time in both groups, as reported in other studies^28^. The rate of tissue repair increased at the experimental times (15, 30, 60 and 90 days). The macroscopically structure and regeneration was similar in both groups, but the healing rate was faster in control group. At 15 days post-operatively there was a greater difference in repair tissue production values between the groups (CG and MG). We believe that this difference is due to the retention of autogenous cortico-cancellous tissue at the bone defect site in the control group, and that these fragments were accounted for as regenerative bone^11^. In the tibias in which demineralized bone matrix was implanted the radiolucent biomaterial filled the bone defect space and no bone fragments were able to occupy the void.

Volumetric parameters achieved from microCT, such as bone volume (BV) and bone volume fraction (BV/TV), allow to evaluate the newly formed bone tissue^27^. In the present study, a significant increase in BV and BV/TV was observed between 15 and 30 after DBMc implantation, demonstrating greater intralesional bone activity and production, as observed previously in a study that compared two human DBMs in rats, with minimal bone production in 2 weeks, subsequently followed by favorable progress in bone density at 4 and 8 weeks^29^. Microstructural analysis parameters such as trabecular thickness (Tb.Th) and separation (Tb.Sp) do not demonstrate statistical variation, leading to an understanding that a similar pattern of trabecular formation was achieved between the groups, and more consistent variation in relation to the trabecular number (Tb.N), with higher values for CG.

At 60 days postoperatively, bridging bone growth was observed between the margins of the bone defects in both groups, corroborating the results of Zhukauskas *et al*.^30^, who demonstrated bridging bone formation six weeks after the application of human demineralized bone matrix in the tibia of rabbits. According to the same authors, after 12 weeks of implantation, the matrix had been completely resorbed and replaced by newly formed bone, similar to the results of our study where at 90 days, the bone defect showed partial filling with new bone tissue in both groups. In a study^31^, it was evaluated the use of hydroxyapatite in rabbit tibial defects, and also obtained bone repair times similar to the present study notably that at 6 weeks following implantation of biomaterials cortical repair was not yet radiographically visible. After 12 weeks, cortical bone formation began, with bone growth occurring from the extremities to the center of the defect, completing the repair after 24 weeks. However, this experimental model has limitations^32^, since even bone defects that did not receive biomaterial showed complete repair in 24 weeks, as observed in our study.

It is likely that the highest bone production observed in the control group is due to the osteogenic, osteoconductive and osteoinductive properties present in autogenous cancellous bone, considered the gold standard bone graft^33,34^, which persisted into the bone defect after drilling it. Similar results were reported in a previous study, where bone defects without filling showed higher repair rates when compared to those ones filled by demineralized allogeneic bone matrix^11,35^. Factors related to the biomaterial may also explain the lower bone production rate observed in the tibias that received the caprine DBM, such as the absence of adequate carrier, granulometry has not been standardized as an evaluation of regeneration and the osteoinductive potential of the DBM^1,26^.

Two limitations of this study can be highlighted. Despite microCT has provided information needed to accomplish the objectives of the present study, a histopathological analysis could have added more accuracy in the assessment of cicatricial process and sensitivity for differentiating tissues, in spite of its two-dimensional feature^36^. Additionally, it is believed that the addition of a carrier and granulometry standardization, could have helped fixing the biomaterial into the receiver bone site and reducing the surface of contact of the biomaterial^5,13,23^.

Our study demonstrated that there was no negative interference from DBMc in the repair of bone defects in rabbits, with microtomographic evidence of bone production in both groups, following similar microstructural pattern compared to the physiological one.

## Conclusions

These results indicate that caprine demineralized bone matrix is bio tolerable and safe. When DBMc is used, animals show early bone repair within 30 days of implantation, and the formation of bone bridge at 60 days. Future studies are needed to establish when bone remodeling starts, as well as the importance of characteristics of the DBMc such as granularity, ideal carrier and possible interactions between caprine demineralized bone matrix and other materials.

## Footnotes

1 Research performed at Laboratory of Bases of Surgical Techniques, Department of Clinical and Veterinary Surgery, Faculty of Agricultural and Veterinary Sciences (FCAV), Universidade Estadual Paulista “Julio de Mesquita Filho”, Jaboticabal-SP, Brazil. Part of Master degree thesis, Postgraduate Program in Veterinary Surgery. Tutor: Prof. Bruno Watanabe Minto.
